# Carbon Nanotubes Filled with Different Ferromagnetic Alloys Affect the Growth and Development of Rice Seedlings by Changing the C:N Ratio and Plant Hormones Concentrations

**DOI:** 10.1371/journal.pone.0157264

**Published:** 2016-06-10

**Authors:** Yi Hao, Feifan Yu, Ruitao Lv, Chuanxin Ma, Zetian Zhang, Yukui Rui, Liming Liu, Weidong Cao, Baoshan Xing

**Affiliations:** 1 College of Resources and Environmental Sciences, China Agricultural University, Beijing100093, People's Republic of China; 2 Key Laboratory of Advanced Materials (MOE), School of Materials Science and Engineering, Tsinghua University, Beijing 100084, People's Republic of China; 3 Stockbridge School of Agriculture, University of Massachusetts, Amherst, MA 01003, United States of America; 4 Institute of Agricultural Resources and Regional Planning, Chinese Academy of Agricultural Sciences, Ministry of Agriculture Key Laboratory of Crop Nutrition and Fertilization, Beijing 100081, China; Chunag-Ang University, REPUBLIC OF KOREA

## Abstract

The aim of this study was to investigate the phytotoxicity of thin-walled carbon nanotubes (CNTs) to rice (*Oryza sativa* L.) seedlings. Three different CNTs, including hollow multi-walled carbon nanotubes (MWCNTs), Fe-filled carbon nanotubes (Fe-CNTs), and Fe-Co-filled carbon nanotubes (FeCo-CNTs), were evaluated. The CNTs significantly inhibited rice growth by decreasing the concentrations of endogenous plant hormones. The carbon to nitrogen ratio (C:N ratio) significantly increased in rice roots after treatments with CNTs, and all three types of CNTs had the same effects on the C:N ratio. Interestingly, the increase in the C:N ratio in roots was largely because of decreased N content, indicating that the CNTs significantly decreased N assimilation. Analyses of the Fe and Co contents in plant tissues, transmission electron microscope (TEM) observations and energy dispersive X-ray spectroscopy (EDS) analysis proved that the CNTs could penetrate the cell wall and the cell membrane, and then enter the root cells. According to the author's knowledge, this is the first time to study the relationship between carbon nanotubes and carbon nitrogen ratio and plant hormones.

## Introduction

First discovered by Lijima in 1991, carbon nanotubes (CNTs) are generally categorized as single-walled carbon nanotubes (SWCNTs) and multi-walled carbon nanotubes (MWCNTs) based on the number of layers of carbon atoms [1–4]. Among various nanomaterials, CNTs are particularly attractive because of their unique chemical, physical, and electrical properties [5,6]. CNTs have great potential for use in numerous applications such as computers, aircraft airframes, and sporting goods. Also, they have emerged as efficient drug delivery carriers in the biomedical field [4, 7]. CNTs filled with ferromagnetic metals show antioxidant properties and long-term stability, and have potential applications in anisotropic magnetic responses, high-density magnetic recording media, and biomedicine [8]. CNTs filled with FeCo have potential uses as lightweight and high-efficiency microwave absorbers [9]. Because of their diverse potential applications, the production of CNTs for use in both industrial and consumers’ products has increased. The production capacity of CNTs was 2500 metric tons in 2010, but was expected to exceed 12,800 metric tons in 2016 [10,11]. As a result, the amount of CNTs released into the environment will inevitably increase. Therefore, it is necessary to investigate and understand the effects of these particles on the ecosystem, including their toxicity to plants [12,13].

Recent studies have shown that the physiological responses to CNTs may be variable or even opposite among different plant species. For example, alfalfa and wheat tolerated high concentrations of industrial-grade CNTs, and even showed enhanced root development [14]. Similarly, MWCNTs accumulated in wheat and rapeseed to less than 0.005‰ of the applied MWCNT dose, and did not have any measurable effects on the plants. These results implied that the amount of MWCNTs entering the food chain via food crops may be very low [15]. In another study, corn, barley, and soybean seeds exposed to MWCNTs showed accelerated germination, and there were no negative effects on plant development [16]. MWCNTs were shown to enhance the growth of tobacco and regulate cell division via activating water channels and regulating genes involved in cell division and extension [17]. Water-soluble CNTs were shown to stimulate the growth of gram (*Cicer arietinum*) plants by increasing their ability to absorb and retain water [18]. Tomato plants grown in soil supplemented with CNTs produced twice as many flowers and fruits as did those grown in control soil [19].

In contrast, other studies have shown that CNTs can negatively affect plant growth. For example, MWCNTs have been shown to affect plants’ phenotypes. MWCNTs inhibited the growth of soybean [20]. Similarly, SWCNTs adversely affected protoplasts of rice and *Arabidopsis*, resulting in programmed cell death [21]. In another study, SWCNTs inhibited the growth of maize root hairs, mainly by decreasing the expression levels of genes controlling root growth [22]. MWCNTs significantly altered cellular morphology, destroyed membrane integrity, and disrupted mitochondrial function in root cells of *Allium cepa* [23]. Many studies have investigated the phytotoxicity of nanomaterials other than CNTs, such as CeO_2_ nanoparticles (NPs) [24–26], La_2_O_3_ NPs [24], ZnO NPs [27, 28], CuO NPs [29], Au NPs [30, 31], Ag NPs [32, 33], Al_2_O_3_ NPs [34], and TiO_2_ NPs [35]. These studies have not only provided meaningful guidance, but also identified which physiological indexes are reliable indicators of plants’ responses to novel nanomaterials.

The C: N ratio of biomass is generally regarded as a good indicator of the relative availability of C and N [36]. This ratio has been used in systematic analyses of the forest floor and topsoil [37], to assess the influence of elevated CO_2_ concentration on environment [38], and as an indicator of secondary compound concentrations in plant organs [36]. A recent study showed that a lower C:N ratio in the leaf was associated with increased leaf Bt protein content in Bt cotton [39]. However, to our knowledge, there is no study on the effects of CNTs filled with different metals or alloys on the C:N ratio of plants.

In this study, the hybrid rice line Y Liangyou 1928 was selected as the model plant. Rice plants were treated with different concentrations of three different CNTs; hollow multi-walled carbon nanotubes (MWCNTs), Fe-filled carbon nanotubes (Fe-CNTs) and Fe-Co-filled carbon nanotubes (FeCo-CNTs). We investigated the phytotoxicity of CNTs to rice seedlings, including effects on growth and development and on C, N, and phytohormone contents. To the best of our knowledge, this is the first report on the effects of CNTs filled with various ferromagnetic alloys on the C: N ratio in plants. This study describes the physiological responses of rice seedlings to several different CNTs. These results will be useful for the sustainable and healthy development of carbon nanomaterials.

## Materials and Methods

### Sample preparation and characterization of the three CNTs

FeCo-filled CNTs were synthesized as described previously [8]. Briefly, ferrocene and cobaltocene powders were dissolved in trichlorobenzene (TCB) (C_6_H_3_Cl_3_), and then injected with a syringe pump into a chemical vapor deposition (CVD) furnace heated to 860°C. Argon and H_2_ were delivered into the furnace at flow rates of 2000 and 3000 sccm respectively. The reaction was allowed to proceed for 30 min before shutting down the CVD furnace and allowing it to cool to room temperature naturally.

Similarly, Fe-filled CNTs were synthesized as described by Lv et al. [9]. Briefly, dichlorobenzene was selected as the solvent to dissolve ferrocene powder. The solution was pumped into a tubular quartz reactor with a reaction temperature of 860°C. At the same time, Ar and H_2_ were delivered to the reactor with flow rates of 2000 and 3000 sccm, respectively. Finally, the Fe-CNTs were peeled off from the inner wall of the reactor.

MWCNTs were provided by the laboratory of Professor Wei Fei (Tsinghua University) and were produced using a nano-agglomerate fluidized-bed reactor [40–42]. All these three carbon nanomaterials were purified before phytotoxicity experiments.

Other chemicals were of analytical grade and were purchased from Beijing Chemical Plant (Beijing, China). A transmission electron microscope (TEM) (JEM-2100, JEOL, Japan) was used to determine the morphology and size of CNTs before use in experiments. To prepare the TEM samples, the CNTs were dissolved and sonicated in ethanol and then dropped onto Cu grids. Raman characterizations were taken by raman spectrometer (LabRAM HR Evolution, HORIBA Jobin Yvon, France) at 532 nm. Spectra of these three carbon nanotubes were collected in the range of 800–3500 cm^-1^ with the scan time settings for 30s.

### Hydroponic rice cultivation

Hybrid rice Y Liangyou 1928 was selected as the test plant to investigate the effects of the three CNTs on seedling growth. Rice seeds (purchased from China Agricultural University, Beijing, China) were sterilized in 5% H_2_O_2_ for 30 min, and then rinsed three times in deionized water. The sterilized seeds were placed on moist filter paper in Petri dishes (100 mm × 15 mm), and the dishes were sealed with parafilm before being placed in an electro-heating standing-temperature cultivator (DRP-9052, Peiyin, China). The seeds were germinated at 25°C in darkness. After 5 days, seedlings with a similar size were selected and transplanted into 50-mL centrifugal tubes containing 45 mL 1/2 strength Kimura nutrient solution, and grown under greenhouse conditions. At 5 day after transplanting, the rice seedlings were treated with the three different carbon nanotubes (MWCNTs, Fe-CNTs, and FeCo-CNTs) at concentrations of 0, 10, 50, and 300 mg/L. Considering precipitation and accumulation of carbon nanomaterials in nutrient solution, all suspensions were sonicated at 25°C for 30 min in a water bath. After transplanting rice seedlings, the suspensions were stirred by a glass rod every 12 hours during exposure. All experiments were carried out in the greenhouse at China Agricultural University in March 2015. Triplicate samples were applied for analyses [43–45].

### Measurements of biomass and root and shoot lengths

After 15 days of exposure to three different carbon nanomaterials, the rice seedlings were thoroughly washed with tap water and then rinsed with deionized water. Root length was defined as the distance from the root tip to the root base. Shoot length was defined as the distance from the leaf base to the leaf tip.

The roots and shoots were separated, and the fresh weights were determined. The samples were dried in a fan-forced oven at 105°C for 20 min, and then at 80°C to stable weight before measuring dry weight [46].

### TEM observations of CNTs in rice seedling roots and EDS spectra

After treatment for 15 days, fresh rice roots treated with MWCNTs, Fe-CNTs, or FeCo-CNTs (0 and 300 mg/L) were thoroughly washed with deionized water. The rice roots were then prepared to observe the localization of the three different CNTs inside the cells. Briefly, root apices were prefixed in 2.5% glutaraldehyde, and then dehydrated in an ethanol gradient series before being embedded in Spurr’s resin [47, 48]. Samples were cut into 90-nm thick sections using a microtome with a diamond knife and collected on Cu-based grids. All samples were observed under a transmission electron microscope (TEM) (JEM-1400, JEOL, Japan) operating at 80 KV. Energy-dispersive X-ray spectroscopy (EDS) was conducted to further confirm the presence of FeCo-CNTs in the rice roots.

### Measurement of Fe and Co contents

After treatment for 15 days, dried shoots and roots of rice seedlings were separated to determine the concentrations of Fe and Co. Samples (50–100 mg) were digested in a mixture of HNO_3_-HF(1:2) for 24 h in a Single Reaction Chamber Microwave Digestion System (MILESTONE, LabTech, Vergamo, Italy). Then, the acid mixture was evaporated by heating the mixture at 210°C on an electric plate (VB20, LabTech) for several hours until the solution reduced to 1 ml. The residue was diluted with ultrapure water, and then the Fe concentration was analyzed by inductively coupled plasma optical emission spectrometry (ICP-OES; ICAP 6300, Thermo Scientific, Waltham, MA, USA). The Co concentration was determined by inductively coupled plasma mass spectrometry (ICP-MS; DRCII, PerkinElmer, Norwalk, CT, USA) [49].

### C: N ratio assay

After exposure for 15 days Dried shoots and roots of rice seedlings were separated to determine C and N contents. After weighing, samples were ground to a powder and then the C and N contents were determined using an elemental analyzer (Vario ELIII, Elementar, Hanau, Germany). The C: N ratio was calculated as follows: C:N ratio = C content*100/N content.

### Phytohormone analyses

After harvesting, Absisic acid (ABA), indole-3-acetic acid (IAA), isopentenyl adenosine (IPA), jasmonic acid (JA), brassinolide (BR), and gibberellic acid 1+3 (GA1+3) were extracted, purified, and quantified by ELISA methods as described in Gawronska *et al*. [50], using reagents and antibodies provided by Professor BaomingWang (China Agricultural University, Beijing, China).

### Data analysis

All experiments were conducted in triplicate. Values shown are mean ± standard deviation (SD). Statistical analyses (one-way analysis of variance, ANOVA, and Dunnett’s test) were conducted using SPSS 19.0 for windows (SPSS, Chicago, IL, USA). In all cases, a value of *p*<0.05 was considered to be statistically significant.

## Results

### Characterization of CNTs

**Fig 1:**
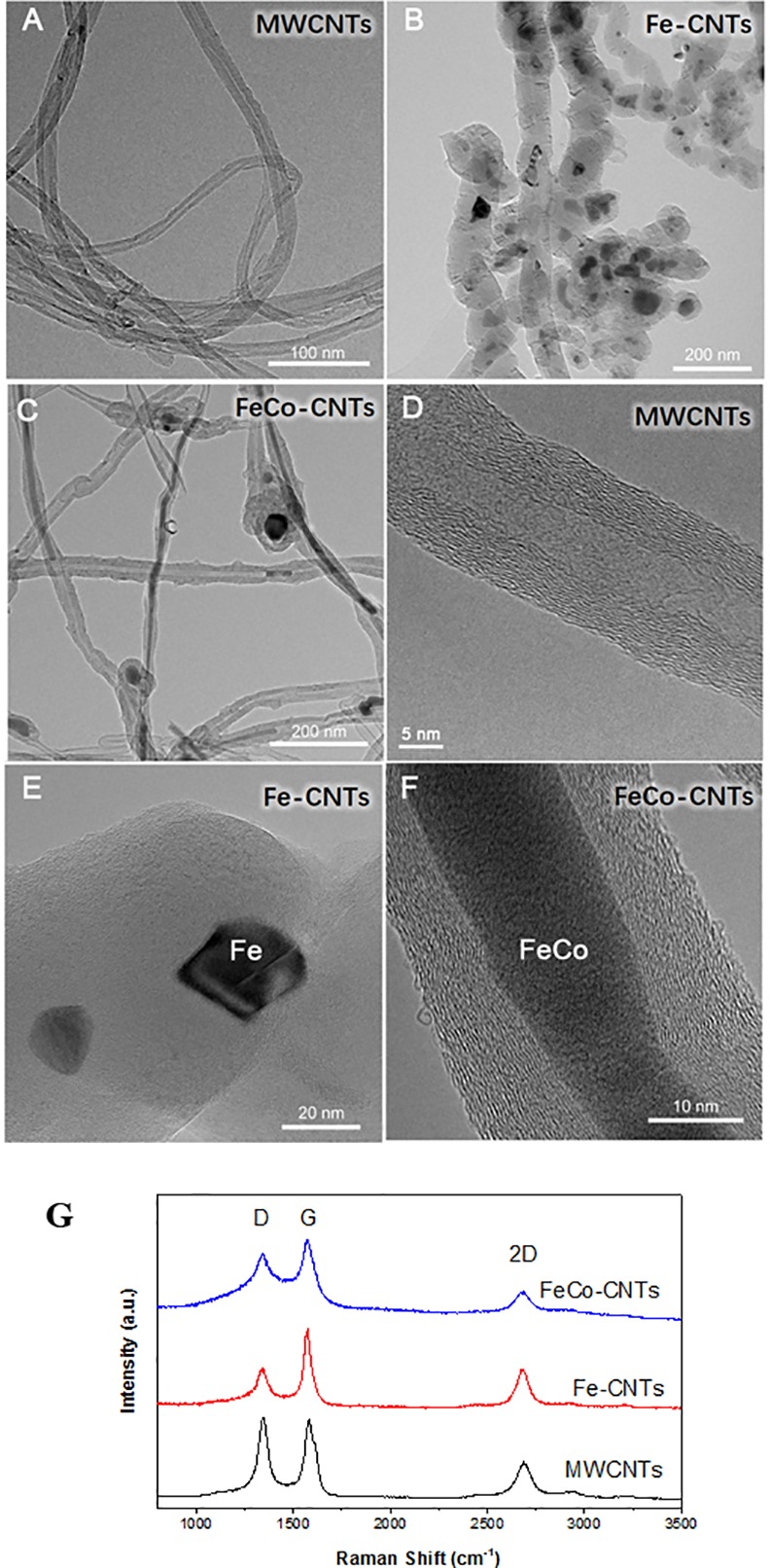
TEM and HRTEM image of MWCNTs (A, D), Fe-CNTs (B, E), and FeCo-CNTs (C, F), and Raman spectra of each CNTs (G).

The typical diameters of MWCNTs, Fe-CNTs, and FeCo-CNTs were dozens of nanometers (Fig 1). Because they are a kind of one-dimensional nanomaterial, the CNTs agglomerated and mingled easily, and readily attached to the edge of the carbon films on the Cu grids. All of the MWCNTs were hollow, continuous and relatively long (Fig 1A and 1D). The typical wall thickness ranged from approximately 5 nm, indicating ~15 carbon atom layers (Fig 1D). The Fe-CNTs had a bamboo-like shape with many joints, and some of the hollow space was filled with Fe (dark areas in Fig 1B and 1E). Some of the Fe-Co CNTs were filled with Fe-Co alloy inside the cavities of the CNTs (Fig 1C and 1F). The high resolution TEM images also showed that all the samples were multiwalls with more than ten carbon layers and the metals were filled into the carbon tubes.

Raman spectra of each sample were showed in Fig 1G. The peaks at ~1342 and ~1580 cm-1 were corresponding to the D and G bands, respectively, and all the spectra were normalized by intensity of G band. D band was often regarded as degree of defects and disorders, and G band was regarded as degree of crystallinity. Intensity ratios (ID/IG) of MWCNTs, FeCo-CNTs, and Fe-CNTs were 1.03, 0.84, and 0.51, respectively. Relatively high degree of defects in MWCNTs might due to the fluidized-bed method and the other two samples might due to the doping of metals.

### Effects of CNTs on the C: N ratio of rice roots and shoots

The C: N ratio in roots was higher in plants treated with carbon nanoparticles, even at low concentrations (10 mg/L), than in untreated (CK) plants. The three kinds of CNTs had the same effects on the C:N ratio of rice roots (Table 1). Notably, the increase in the C: N ratio in roots was largely because of decreased N content, indicating that the CNTs significantly decreased N assimilation.

**Table 1:** C: N ratio in rice roots and shoots after treatments with three different carbon nanotubes.

		Roots				Shoots			
	Concentration (mg/L)	0	10	50	300	0	10	50	300
MWCNTs	C contents (%)	44.24a	44.49a	43.20b	43.86ab	42.06a	41.49ab	41.19b	41.68ab
	N contents (%)	1.09a	0.95b	0.87bc	0.76c	1.86b	1.78b	2.15a	1.90ab
	C:N Ratio	40.48c	46.93bc	49.93ab	57.64a	22.50a	23.35a	18.68b	21.91ab
Fe-CNTs	C contents (%)	44.24b	44.44b	44.24b	45.46a	42.06a	42.62a	41.96a	42.91a
	N contents (%)	1.09a	0.79b	0.78b	0.76b	1.87ab	1.97a	1.71b	1.90ab
	C:N Ratio	40.48b	56.46a	56.56a	59.82a	22.50ab	21.66b	24.47a	22.59ab
FeCo-CNTs	C contents (%)	44.24b	45.53a	45.11a	45.41a	42.06a	42.78a	42.86a	42.79a
	N contents (%)	1.09a	0.86b	0.83b	0.82b	1.87a	1.71b	1.66b	1.97a
	C:N Ratio	40.48b	52.70a	54.11a	55.45a	22.50b	25.06a	25.83a	21.69b

Note: Within each data, different letters represent significant difference (p<0.05), compared with control group.

### Fe, Co content in rice roots and shoots after treatments with Fe-CNTs and FeCo-CNTs

**Fig 2:**
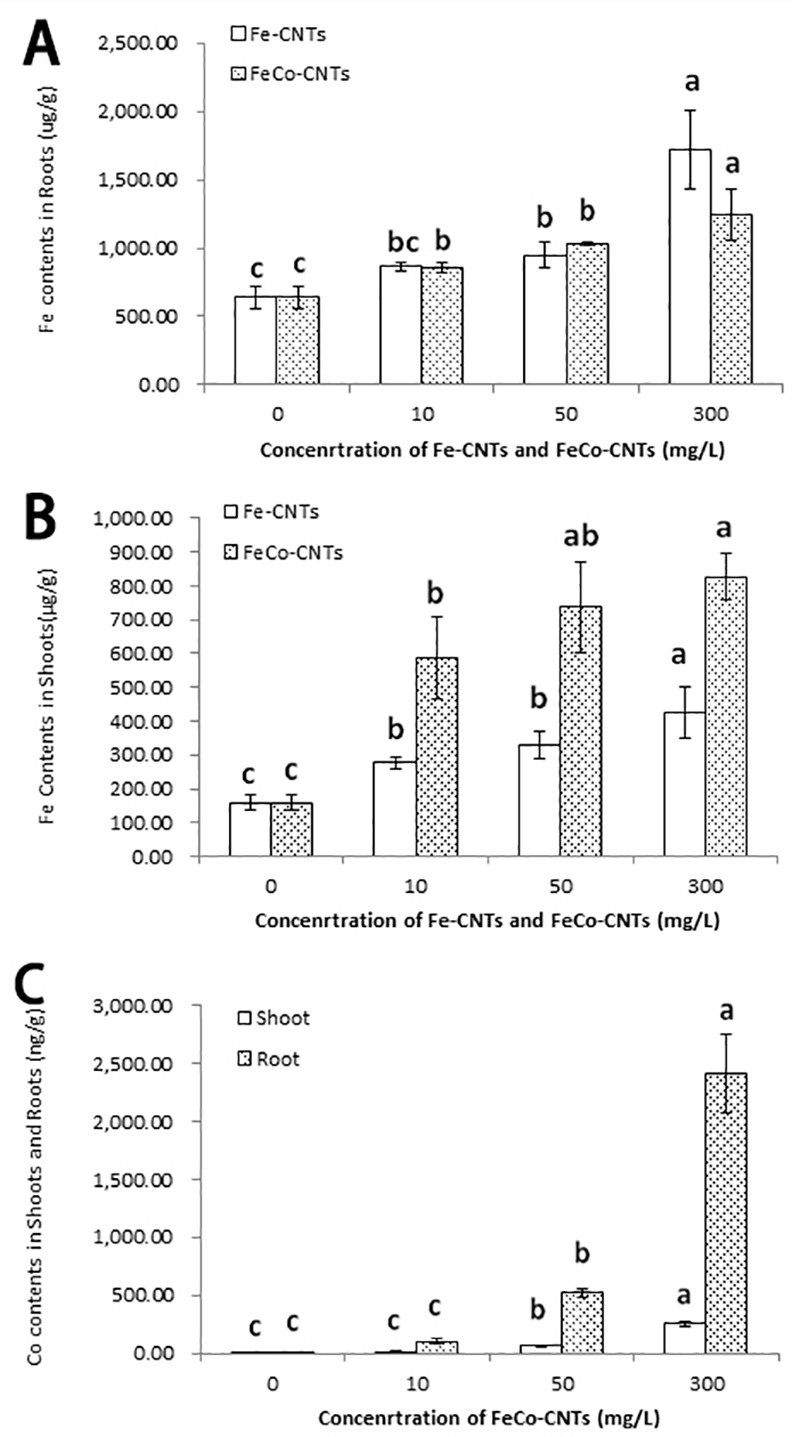
Fe content in rice roots and shoots after treatments with Fe-CNTs (A), FeCo-CNTs(B). and Co contents in rice shoots and rice roots after treatments with FeCo-CNTs(C). Data are average of three replicates ±SE. Bars with different letters are statistically different at p<0.05, compared with control group.

The concentration of Fe, a constituent of Fe-CNTs and FeCo-CNTs, significantly increased in rice roots and rice shoots after treatments with these two CNTs. After treatments with Fe-CNTs and FeCo-CNTs, the Fe concentration was higher in roots than in shoots, especially at low nanoparticle concentrations (10 mg/L) (Fig 2A and 2B).

Similar to the changes in Fe contents in rice roots and shoots, the Co contents increased in roots and shoots after treatments with FeCo-CNTs, especially at concentrations higher than 50 mg/L. (Fig 2C).

### Effects of CNTs on biomass of rice roots and shoots

**Fig 3:**
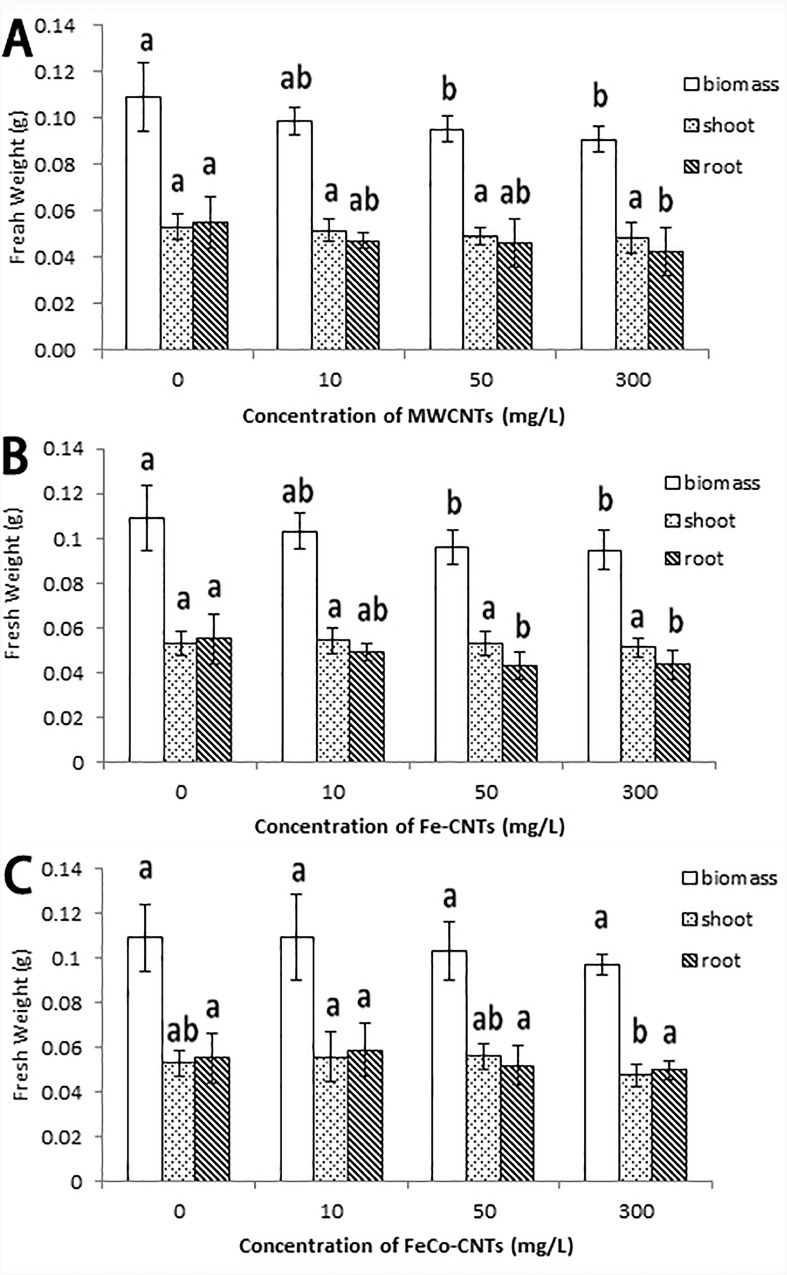
Biomass production of rice after treatments with MWCNTs (A),Fe-CNTs (B) and FeCo-CNTs (C). Data are average of three replicates±SE. Bars with different letters are statistically different at p<0.05, compared with control group.

The biomass production rate (fresh weight) significantly decreased after treatments with MWCNTs and Fe-CNTs at high concentrations (50 mg/L and 300 mg/L; Fig 3A and 3B). The root and shoot biomass showed that the lower biomass was caused by the deceases in root biomass. That is, the MWCNTs and Fe-CNTs first inhibited the growth and development of roots. The effects of FeCo-CNTs on rice biomass production were not significant (Fig 3C).

### Effects of CNTs on rice root length and shoot height

**Fig 4:**
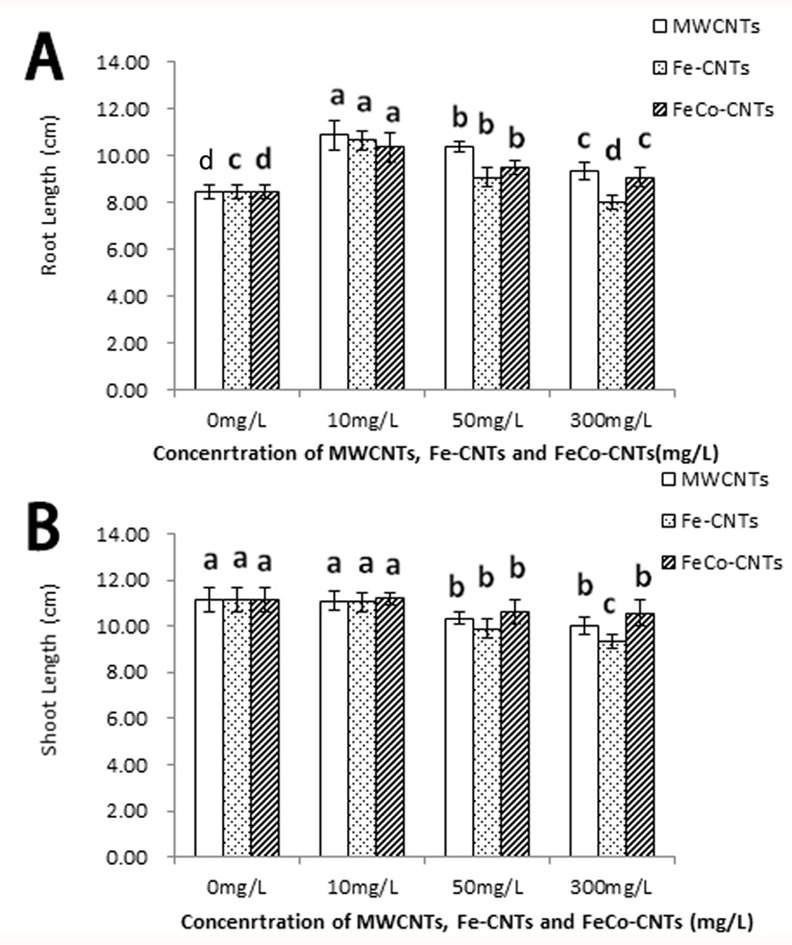
Rice root length (A) and shoot length (B) after treatments with three types of carbon nanoparticles. Data are average of three replicates±SE. Bars with different letters are statistically different at p<0.05, compared with control group.

All three types of CNTs significantly promoted the elongation of rice roots at low concentrations of 10 and 50 mg/L (Fig 4 and S1 Fig). However, the promoting effect became weaker as the CNT concentrations increased. At 300 mg/L, Fe-CNTs nanoparticles inhibited root elongation (Fig 4A). The biomass of rice seedlings was not affected by CNTs at 10 mg/L and was significantly decreased by CNTs at 50 mg/L.

The shoot length response to CNTs differed from the root length response. At high concentrations (50 mg/L and 300 mg/L), all three CNTs significantly inhibited rice shoot growth (Fig 4B). Shoot growth was more strongly inhibited by Fe-CNTs than by the other two types of CNTs. The effects of the CNTs on shoot length were consistent with the decrease in shoot biomass.

### TEM observation of CNTs in rice seedlings and EDS analysis

**Fig 5:**
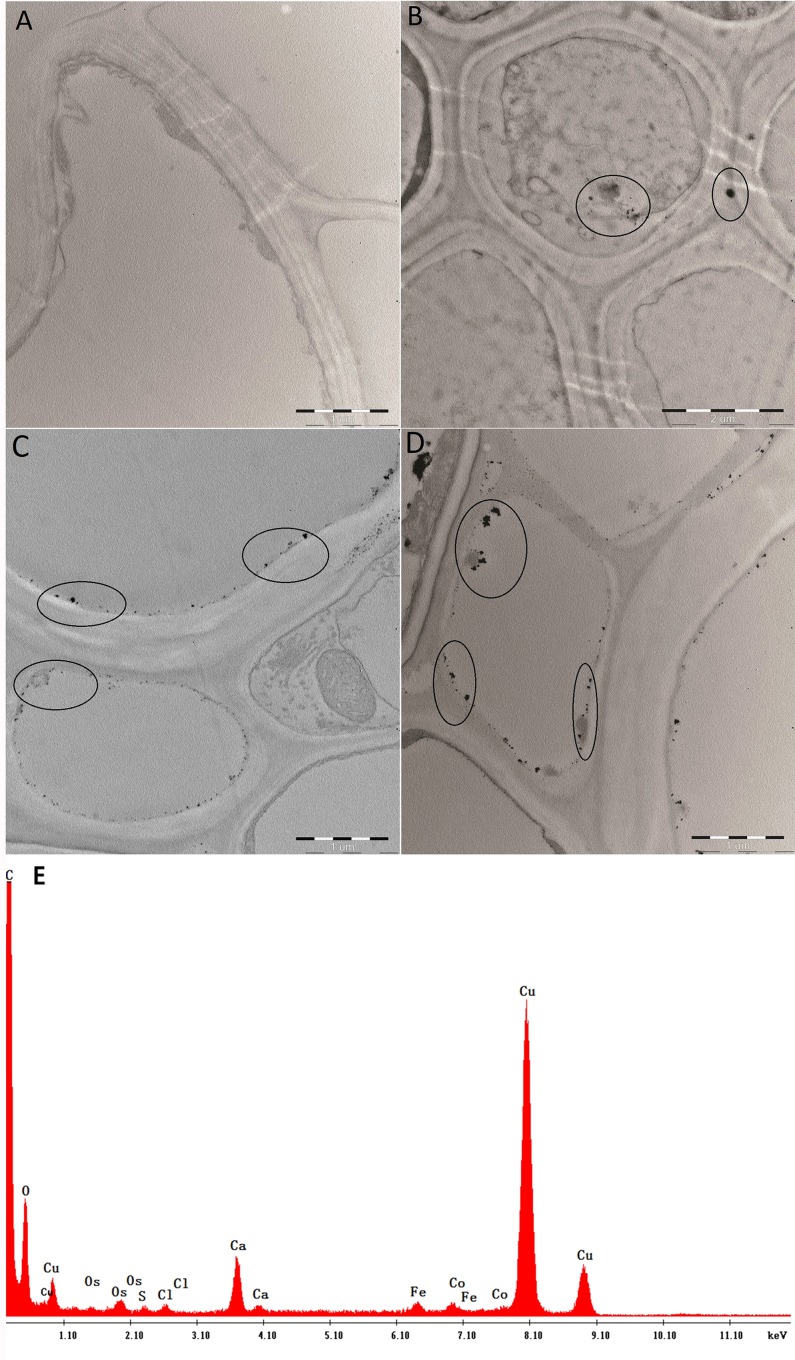
Transmission electron micrographs of untreated rice roots (A), rice roots treated with MWCNTs (B), Fe-CNTs (C), FeCo-CNTs (D), EDS spectra of FeCo-CNTs in rice roots (E). CNTs are circled in images, cw: cell wall.

TEM technique was conducted to localize CNTs in the roots (Fig 5A–5D). There was no CNTs existence in the control treatment (Fig 5A). Nanoparticles were observed in rice root cells by TEM after treatments with MWCNTs, Fe-CNTs and FeCo-CNTs (Fig 5B–5D). The MWCNT nanoparticles were evenly distributed in the cell, but Fe-CNT and FeCo-CNT nanoparticles were distributed around the cell membrane, with some in the cell wall.

Compared with FeCo-CNT particles, Fe-CNT particles were much smaller and showed a more uniform distribution in root cells (Fig 5B–5D). In the study that reported by Le et al. (2015), the Fe-CNT particles were located around organelles or the nucleus, which showed an irregular morphology, consistent with observations of chloroplasts. When the roots accumulated high levels of FeCo-CNT nanoparticles, the root catheter and the root cells became severely deformed (Fig 5D).

Observations of root sections indicated that the thin-walled carbon nanotubes filled with ferromagnetic alloy can penetrate the cell wall and cell membrane to enter cells, from which they can be transported to shoots. However, the three types of CNTs showed different distribution patterns and particle sizes in the plant cells. This could explain the reason that the three types of CNTs had different biological effects.

To further determine the black dots observed by TEM were the CNTs used in rice seedlings cultivation, we used EDS analysis to confirm their elementary composition. The seedlings treated with FeCo-CNTs were selected as test samples considering the low background values of element Co in normal rice cells. The circled area at the top left of Fig 5D was selected to conduct the EDS analysis.

In the EDS spectra of FeCo-CNTs in rice roots (Fig 5E), the obvious peaks of elements Fe and Co were found, which demonstrated the small dots were FeCo-CNTs definitely.

### Effects of carbon nanotubes on phytohormone concentrations in rice seedlings

**Fig 6:**
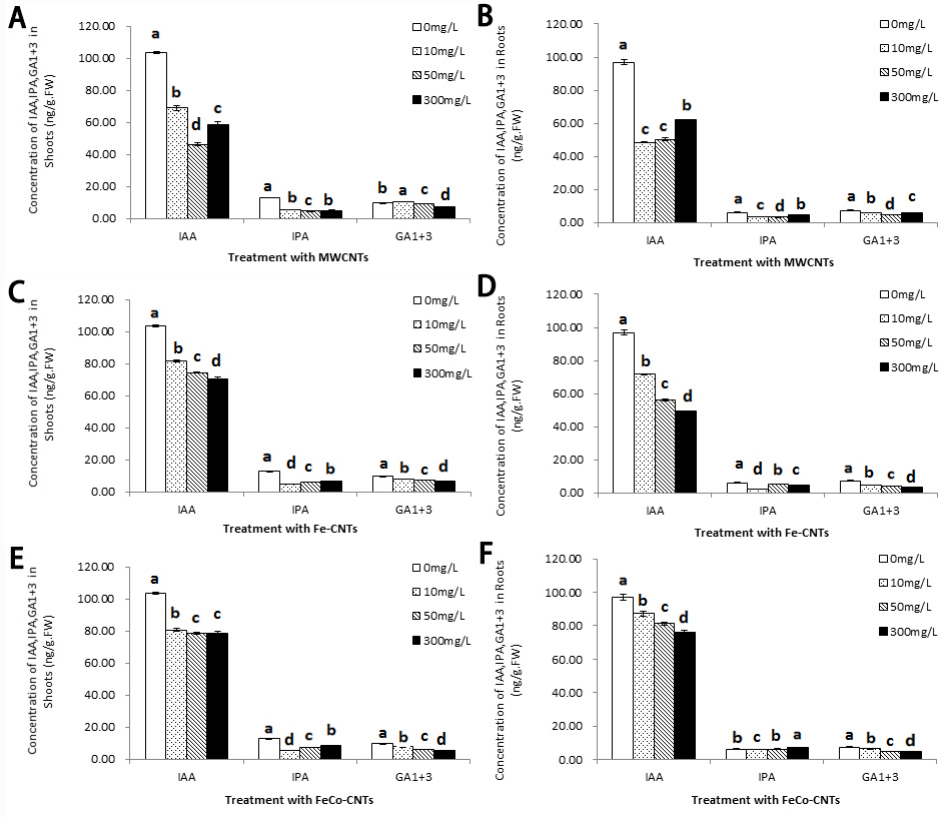
Concentrations of IAA, IPA, and GA1+3 in shoots and roots or rice seedlings after treatments with MWCNTs (A, B), Fe-CNTs (C,D), FeCo-CNTs (E, F). Data are average of three replicates ±SE. Bars with different letters are statistically different at p<0.05, compared with control group.

**Fig 7:**
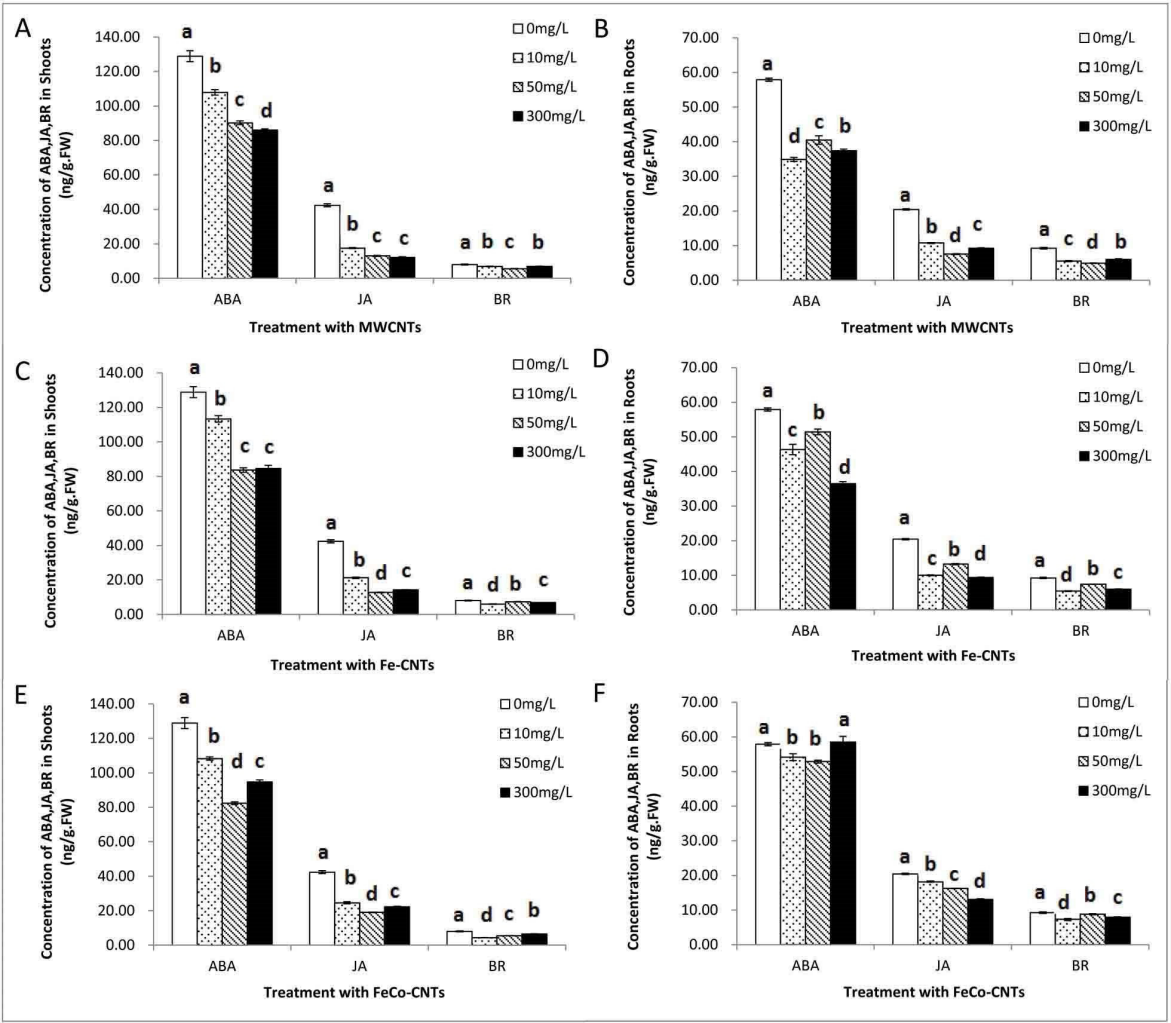
Concentrations of ABA, JA, BR in shoots and roots of rice plants after treatments with MWCNTs (A, B), Fe-CNTs (C, D), FeCo-CNTs (E, F). Data are average of three replicates ±SE. Bars with different letters are statistically different at p<0.05, compared with control group.

The concentrations of auxin (IAA) in rice roots and rice shoots decreased upon exposure to each of the three CNTs at all three concentrations (10, 50, and 300 mg/L) (Fig 6A and 6B). Similarly, other phytohormone including gibberellin (GA1+ 3), cytokinin (IPA) (Fig 6C–6F), jasmonic acid (JA), brassinolide (BR), and absisic acid (ABA) (Fig 7A–7F) showed the same pattern as compared with the control group.

## Discussion

In plants, carbon nutrition usually comes from the atmosphere, but in treatments with carbon nanoparticles, the nanoparticles provide a carbon source because they can be absorbed through the cell membrane. The C:N ratio in roots was higher in plants treated with carbon nanoparticles compared to untreated (CK) plants, and had the same effects on the C:N ratio of rice roots under three kinds of CNTs treatment (Fig 1A–1C).

It is often thought that greater uptake of carbon nanoparticles results in an increased C: N ratio. Interesting, the increased C: N ratio in roots was largely due to decreased N content. The increase in C content, if any, was much less than the decrease in N content. In most treatments, the CNTs had no significant effects on the C:N ratio of rice shoots, and the C and N contents in shoots were not significantly affected by the three CNTs at almost all concentrations. Together, these results indicated that nanotubes only significantly affected the C: N ratio and N assimilation of rice roots. The root elongation was evident in all treatments; this accelerated effect may explain the decrease of N concentration in treated rice roots. Rice roots are the vital organs that absorb water and mineral elements, also have play key roles in assimilation, transformation and synthesis of amino acids and plant hormones[51]. Aligned with our study, CNTs could enhance root growth by manipulating up-regulation of the genes encoding cell division and water channel[16, 19]. Upon exposure to MWCNTs, the rapid growth of rice roots consumed nitrogen and showed the significant decrease of N concentration in roots. This bio-effect may be related to the change of root activities. The underlying mechanism needs to be further investigated.

The element of Fe, a constituent of Fe-CNTs and FeCo-CNTs, significantly increased in rice roots and rice shoots after treatments with these two CNTs, which confirmed that these CNTs, at all concentrations, were absorbed into rice roots and shoots. Meanwhile the Fe content in rice roots and shoots increased with the increasing concentrations of Fe-CNTs and FeCo-CNTs. Notably, the Fe concentration was higher in roots than in shoots, especially at low nanoparticle concentrations (10 mg/L) (Fig 2A and 2B). The increased Fe concentrations in rice different tissues confirmed that the Fe-CNTs and FeCo-CNTs were absorbed into roots and transported to shoots. Comparing with the Fe contents in rice roots and shoots, Co contents increased in roots and shoots after treatments with FeCo-CNTs, especially at concentrations higher than 50 mg/L. These data further indicated that FeCo-CNTs could be taken up into cells and transported within the plant body. The uptake and transport rates were higher with increasing concentrations of FeCo-CNTs (Fig 2C).

The significant inhibition of Fe-CNTs to rice growth in our study could be explained from the aspect of its structural specialties. On the one hand, the nano-filler was differed from other two carbon nanotubes. The existence of Fe nanowires located in the center of carbon nanotubes may inhibit the growth of rice. On the other hand, filled with Fe nanowires, the structure of thin CNTs was changed inevitably. The carbon walls were approximately 5 nm thick. Meanwhile, almost all the carbon nanotubes are filled with Fe nanowires with 2μm in length, 20 nm in diameter (Fig 2). Thus, the structure of plentiful Fe nanowires encapsulated by thin carbon nanotubes (around 5nm thick) seemed to have higher phytotoxicity to rice seedlings, which inhibited the growth of rice shoots.

Transmission electron microscope (TEM) observations indicated that the thin-walled carbon nanotubes filled with ferromagnetic alloy can penetrate the cell wall and cell membrane to enter cells, from which they can be transported to shoots. However, the three types of CNTs showed different distribution patterns and particle sizes in the plant cells. This may explain why the three types of CNTs had different biological effects. Energy dispersive X-ray spectroscopy (EDS) analysis further proved the small dots observed by TEM were the CNTs absorbed by rice roots during the treatment.

The relationship between nanomaterials’ size and phytotoxicity had been widely reported. However, studies on size-based phytotoxicity still lag behind. Generally, uptake and translocation of MWCNTs in plants were closely related to the diameter and length of materials [20]. MWCNTs with similar sizes tended to accumulate in the same types of cells in maize and soybean [20]. Serag *et al*. indicated that short MWCNTs with short length (in a range of 30 to 100 nm in length) tended to target the nucleus, plastids, and vacuoles, which further revealed the close relationship between MWCNTs size and phytotoxicity from the perspective of plant cell biology [52].

Plant hormones regulate many processes in plant growth and development, so the effects of nanotubes on plant hormones should be an important index of toxicity. Gibberellin (GA) is widely regarded as the plant regulation of growth and development, such as breaks dormancy, promotes germination [53], stimulated stem elongation and leaf expansion [54, 55]. Indole-3-acetic acid (IAA) is the most common auxin in plants, which mediates plants growth and development, and also has the function of stress resistance, especially metal stress, such as aluminum and cadmium [56,57,58], it can also alter the expression of relative genes to inhibit hypersensitive response[59]. These three plant hormones are plant hormones that promote plant growth and development. After exposure to each of the three CNTs at all three concentrations (10, 50, and 300 mg/L) the concentrations of IAA, GA1+3, and IPA in rice roots and rice shoots decreased (Fig 6), which indicated that the CNTs could inhibit plant growth, consistent with their effects on biomass and plant height.

Absisic acid(ABA) is an universal sesquiterpenoid plant hormone and has effect on growth and development of plants, such as seed germination and abundance of proteins [60]. It could also mediate abiotic stress responses including drought and high salinity via alteration related genes, regulation of stomatal function and antioxidant defense system [61, 62]. Brassinolide (BR) is a vital steroid hormone that regulate plant growth and development, such as stem and root growth, and the development of flowers and fruits [63].Moreover BR could also regulate the response to biotic and abiotic stresses, it could enhance tolerance to cold [64], drought stress [65], alleviate the salinity injuries with high biological activity [66,67]. Jasmonic acid (JA) is another major plant hormone that mediates abiotic stress responses, growth and development, and pathogens defenses [68, 69]. Many investigations have revealed that JA could increase plant tolerances of cold and freezing [70], regulate salt tolerance and heat tolerance positively [71,72], and improve drought tolerance together with ABA in some cases [73]. All these three phytohormones can improve the stress tolerance of plants. Recently, BR and JA have been regarded as the sixth and seventh plant hormones. These three CNTs significantly decreased the concentrations of ABA, BR, and JA in rice roots and rice shoots (Fig 7). Decreased concentrations of ABA, BR and JA can weaken plants’ tolerance to environmental stress, further indicating that CNTs had toxic effects on rice plants. The CNTs had stronger negative effects on JA concentration than on ABA and BR concentrations.

Notably, the bio-effects of CNTs greatly varied on plant species, even at different stages in the same species. At the seedling stage, CNTs promoted the root elongation of cucumber (*Cucumis sativus*) and onion (*Allium cepa*), while significantly inhibited the roots growth of tomato (*Lycopersicon esculentum*) [74]. There was no obvious effect on root length of cabbage (*Brassica oleracea*) and carrot (*Daucus carota*) [74]. Root length and biomass of wheat (*Triticum aestivum*) seedlings were promoted by oxidized MWCNTs, which could stimulate the increase of cell elongation and dehydrogenase activities in roots [75]. Similarly, this positive effect was also found in the process of mustard (Brassica juncea) growth upon exopsure to oxidized MWCNTs [76]. MWCNTs had no significant effects on tomato height and leaves number during flowering stage, but indeed accelerated the growth of tomato during flowering and mature stages. More than twice flowers and fruits in the MWCNTs amended soil was evident relative to the natural soil [19]. Lahiani et al. reported that MWCNTs accelerated the germination of three crop species, including corn, barley, and soybean, but had no obvious toxic effect on plant growth during the seedling [16]. Reverse transcription polymerase chain reaction (RT-PCR) analysis showed that this positive effect on seed germination is related to regulations of aquaporin genes, which can improve the activity of water channels [16]. We have focused on the effects of different MWCNTs on rice seedling in this study. Similar to the most investigations, rice root length was promoted by three different carbon nanotubes, except for the treatment of Fe-CNTs at highest concentration (300mg/L). The excess amounts of element Fe in rice roots might result in the significant inhibition of root length. The concentration of element Fe was as high as 1719 μg/g in rice roots, which was almost three times than the one in the control group and significantly higher than the ones in all other treatments as well. Different from previous study on wheat, the rice biomass was significantly decreased in the presences of MWCNTs and Fe-CNTs. This converse phenomenon also suggests that the MWCNTs effects may also depend on plant species. In summary, these different effects of MWCNTs on different plant species and different growth stages showed the complexity and diversity of nano-phytotoxicity, also indicated that the long-term study should be conducted under the realistic environment in order to better understand the mechanism on how MWCNTs impact on crop plant growth in terms of grain yield, food quality, and food security.

## Conclusions

Our results showed that three types of nanoparticles (MWCNTs, Fe-CNTs and FeCo-CNTs) had toxic effects on rice seedlings, and inhibited the growth and development of roots and shoots. The C:N ratio in rice roots significantly increased after treatments with CNTs, and all three types of carbon nanoparticles had the same effect. Interestingly, the increase in the C:N ratio in roots was largely due to decreased N content, indicating that the CNTs significantly decreased N assimilation. The CNTs filled with ferromagnetic alloys inhibited rice growth by decreasing the concentrations of all endogenous plant hormones. Analyses of Fe and Co contents and TEM observations showed that the CNTs penetrated the cell wall and cell membrane to enter the cell, where they could be transported to shoots. The three types of CNTs showed different distribution patterns and particle sizes inside the cell, explaining why they had different biological effects. According to the author's knowledge, this is the first time to study the relationship between carbon nanotubes and carbon nitrogen ratio and plant hormones.

## Supporting Information

S1 Fig Phenotypic images of treated rice seedlings. Phenotypic images of rice seedlings treated with different concentrations of MWCNTs (A), Fe-CNTs (B), and FeCo-CNTs (C).
(DOCX)
