# Supplementary material for: Carbon Nanotubes Filled with Different Ferromagnetic Alloys Affect the Growth and Development of Rice Seedlings by Changing the C:N Ratio and Plant Hormones Concentrations
Source: PLoS One. 2016 Jun 10;11(6):e0157264. doi: 10.1371/journal.pone.0157264 (PMC4902202; doi:10.1371/journal.pone.0157264)
Supplement: S1 Fig — Phenotypic images of rice seedlings treated with different concentrations of MWCNTs (A), Fe-CNTs (B), and FeCo-CNTs (C). (DOCX) [file pone.0157264.s001.docx]

**Carbon nanotubes filled with different ferromagnetic alloys affect the growth and development of rice seedlings by changing the C:N ratio and plant hormones concentrations**

**Yi Hao ^1#^, Feifan Yu^2#^, Ruitao Lv ^2^, Chuanxin Ma ^3^, Zetian Zhang^1^, Yukui Rui^1,3^*****, Liming Liu^1^,Weidong Cao^4^**, **and Baoshan Xing^3^**

^1^ College of Resources and Environmental Sciences, China Agricultural University, Beijing100093, People's Republic of China

^2^ Key Laboratory of Advanced Materials (MOE), School of Materials Science and Engineering, Tsinghua University, Beijing 100084, People's Republic of China

^3^ Stockbridge School of Agriculture, University of Massachusetts, Amherst, MA 01003, USA

^4^ Institute of Agricultural Resources and Regional Planning, Chinese Academy of Agricultural Sciences, Ministry of Agriculture Key Laboratory of Crop Nutrition and Fertilization, Beijing 100081, China

^#^These authors contributed equally to this work

Tel: 86-10-62733470

Fax: 86-10-62733470

E-mail: ruiyukui@163.com; [yukuirui@umass.edu](mailto:yukuirui@umass.edu)

Number of Pages: 2

Number of Figures: 1

Number of Tables: 0

Figure S1. Phenotypic images of rice seedlings treated with different concentrations of MWCNTs (A), Fe-CNTs (B), and FeCo-CNTs (C).


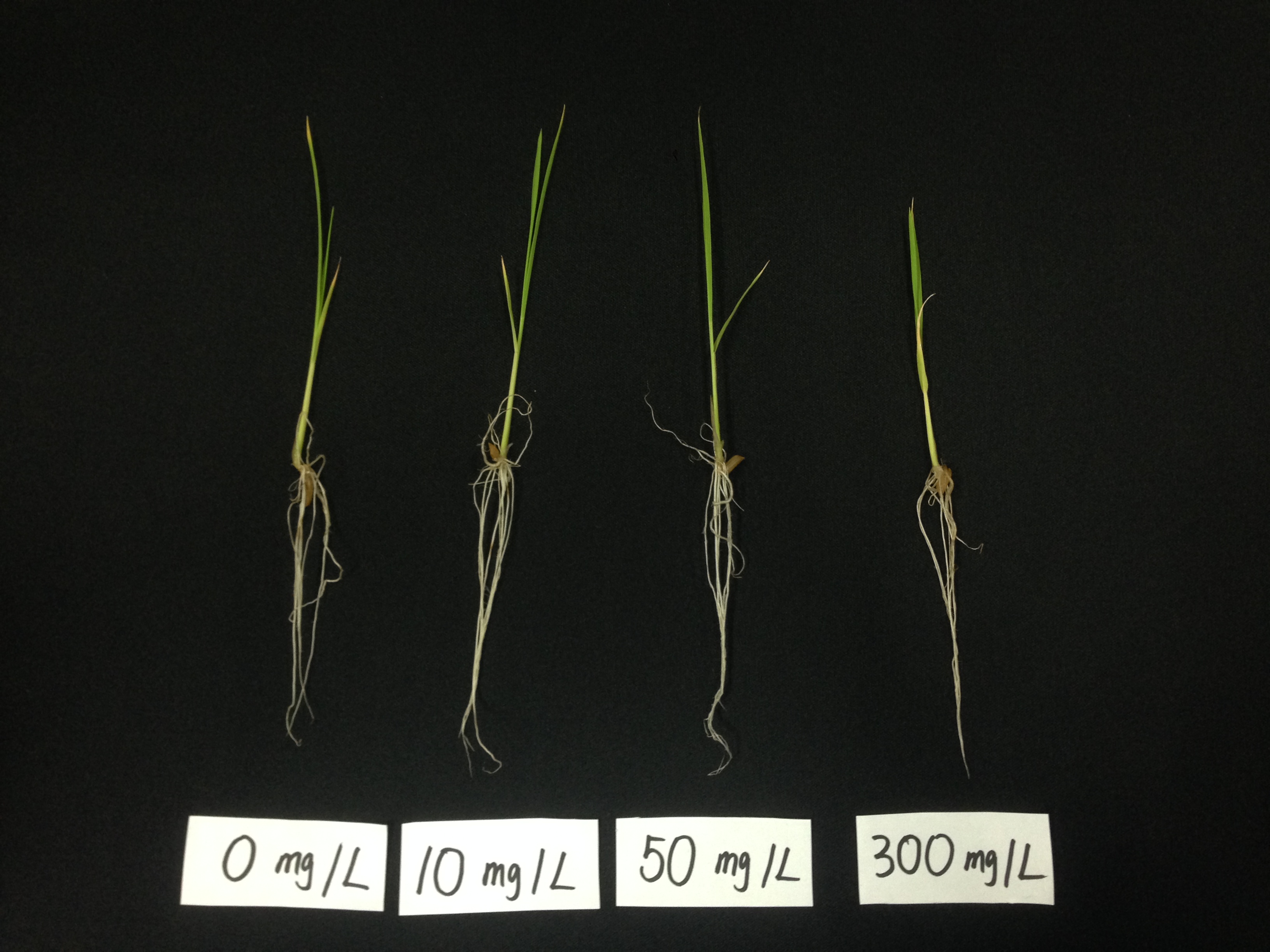

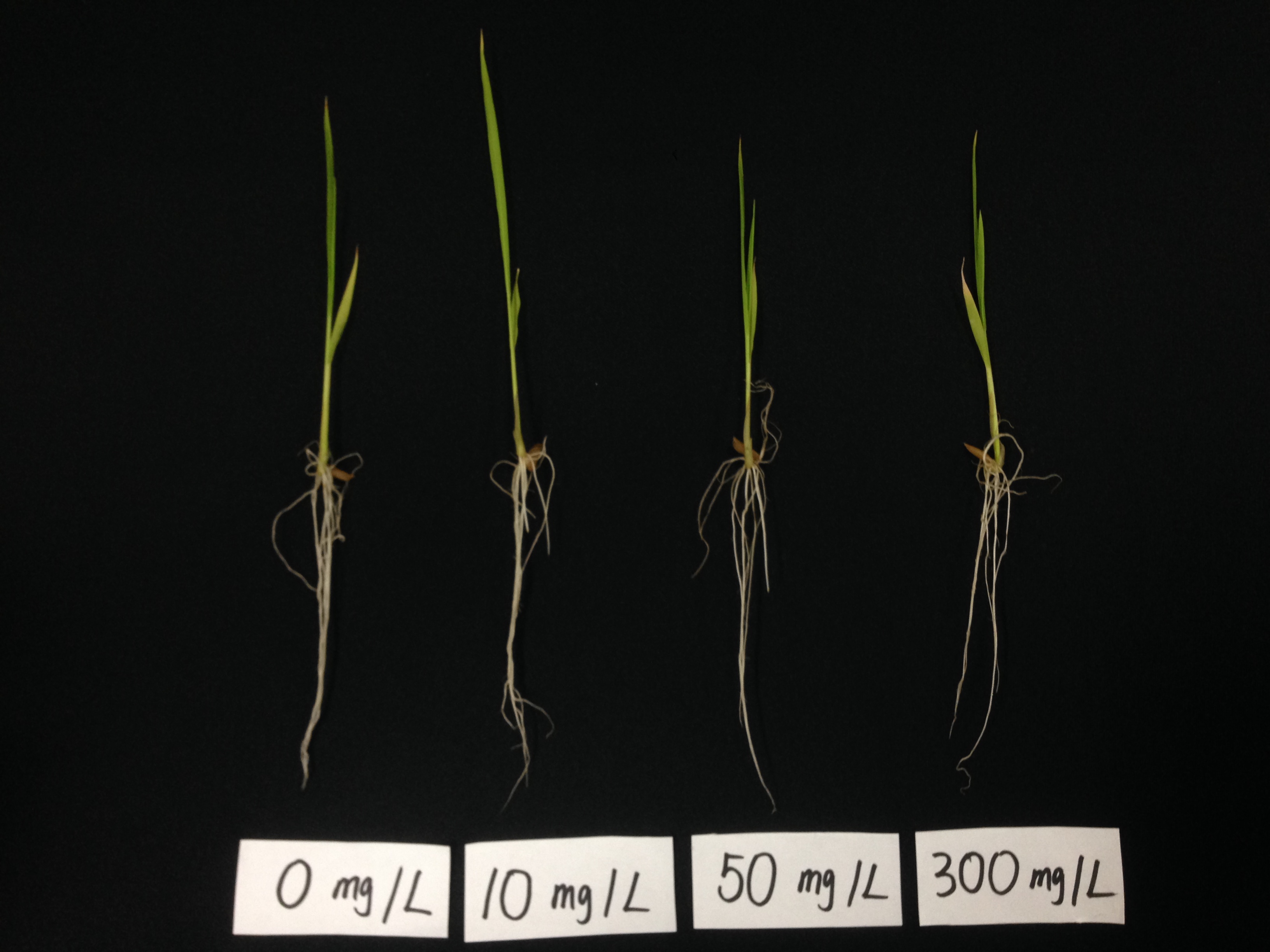

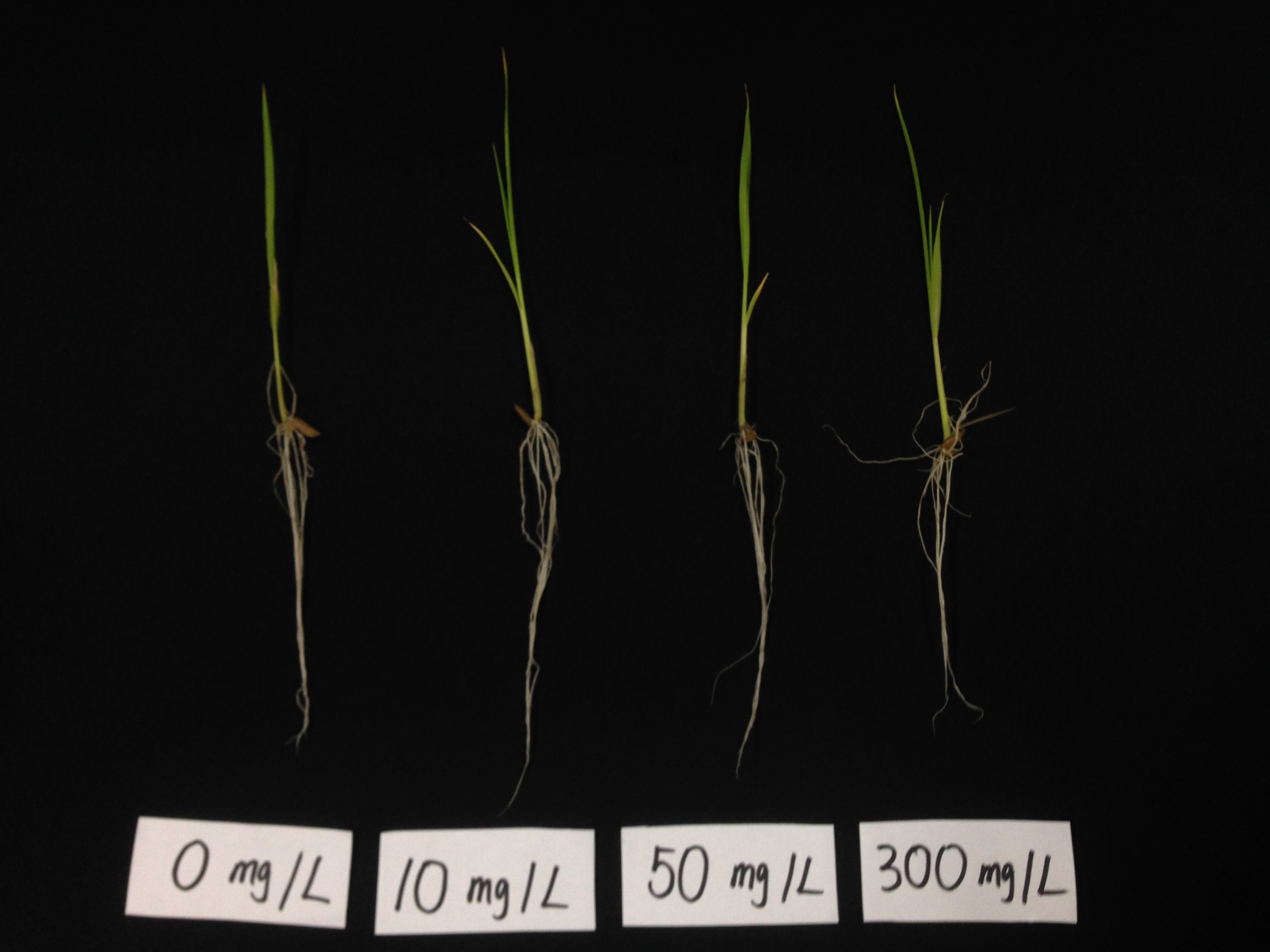


A

BA

C
